# Factors Influencing Interest and Engagement in Biomedical Research Among Community Medicine Residents in India: A Descriptive Cross-Sectional Study

**DOI:** 10.7759/cureus.64831

**Published:** 2024-07-18

**Authors:** Nurul Haque Siddiqui, Richa Mishra, Harish C Tiwari, Imran Ahmed Khan

**Affiliations:** 1 Anaesthesiology, Balrampur Hospital, Lucknow, IND; 2 Community Medicine, Mahamaya Rajkiya Allopathic Medical College, Ambedkarnagar, IND; 3 Community Medicine, Baba Raghav Das Medical College, Gorakhpur, IND

**Keywords:** research methodology, health research, community medicine, biostatistics, biomedical research

## Abstract

Introduction

Medical science must be based on sound and scientific evidence and requires continuous research. Engaging in research allows students and faculty to explore new frontiers, question existing paradigms, and discover innovative solutions to medical challenges. As a specialty, community medicine plays a pivotal role in addressing public health issues. However, the engagement of community medicine residents in biomedical research remains suboptimal, which may impede the generation of evidence-based practices tailored to the Indian context. This study was conducted to find the interest and engagement of community medicine residents, and factors influencing their interest in biomedical research.

Methods

An online survey was conducted among community medicine residents of Uttar Pradesh, from February to April 2024, using Google Forms having a semi-structured, pretested questionnaire.

Results

One hundred and ninety-six residents participated in the study, where females (52.6%; 103/196) outnumbered males (47.4%; 93/196). The majority of participants were third-year residents (40.8%). Most participants seemed interested in biomedical research (83.2%) and thought that Basic Course in Biomedical Research (BCBR) helps conduct research projects (75%). Around half had previous experience in research projects, with cross-sectional studies being the most common (75.9%) study design. Enhancing research skills and a desire to contribute to medical knowledge emerged as primary motivators. On the other hand, the lack of time due to being overburdened with academic and educational activities was seen as the most common barrier to conducting research.

Conclusions

The majority of participants were found interested in research activities. The opportunity to improve research skills, desire to serve the medical fraternity, and a positive impact on resumes were the leading motivating factors for conducting research. Difficulty in sparing time, little knowledge, and poor support from mentors were found as important barriers.

## Introduction

Medical science must be based on sound and scientific evidence and requires continuous research. Engaging in research contributes to the continuous evolution of medical knowledge [1]. It allows students and faculty to explore new frontiers, question existing paradigms, and discover innovative solutions to medical challenges. This process not only advances the field but also nurtures a spirit of inquiry and lifelong learning among medical professionals. Research also plays a key role in the promotion of evidence-based medicine, as students learn to critically appraise scientific literature and incorporate the latest research findings into clinical decision-making. Furthermore, involvement in research develops critical thinking abilities and problem-solving skills. Evidence suggests that students do not realize the importance of research for clinical practice until the clinical phase of medical training when they encounter real-life problems in patient care [2]. Research experience prepares medical students for leadership roles in academia, healthcare administration, and policymaking, empowering them to drive positive change and contribute to medical advancements.

The healthcare system in India is unique due to its diverse population, varying socioeconomic backgrounds, and a wide spectrum of health challenges. As a specialty, community medicine plays a pivotal role in addressing public health issues. It provides promotive, preventive, therapeutic, and rehabilitative care [3]. It also helps implement health policies at the grassroots level. The curriculum of community medicine prepares them to deliver these services. However, the engagement of community medicine residents in biomedical research remains suboptimal, which may impede the generation of evidence-based practices tailored to the Indian context. Understanding these factors is crucial for developing strategies to inculcate a robust research culture among community medicine residents.

Previous studies have highlighted the barriers to research participation among medical professionals, such as lack of time, inadequate research training, and limited access to resources [4,5]. However, limited comprehensive studies are focusing specifically on community medicine residents in India [6]. This gap in the literature necessitates an exploration of the various factors that influence their interest and engagement in biomedical research.

This study was conducted with the objectives to study the interest and engagement of community medicine residents in research and publication and to explore factors influencing their interest in doing biomedical research. By examining variables such as demographic characteristics, institutional support, previous exposure to research, and personal attitudes toward research, this study seeks to provide insights that could guide policy changes and educational interventions. Ultimately, the goal is to enhance research capacity and promote a culture of inquiry within the community medicine discipline, thereby contributing to the advancement of healthcare in India and the achievement of universal health coverage.

## Materials and methods

Type of study

This study used an online survey through Google Forms (Google LLC, Mountain View, California, United States) using a semi-structured, pretested questionnaire.

Study participants

All postgraduate community medicine residents of Uttar Pradesh were eligible to participate in this survey.

Study tool

We compiled a semi-structured questionnaire based on our study objectives, taking guidance from previous studies [7,8].

Methodology

This online survey was conducted from February to April 2024 among community medicine residents of Uttar Pradesh through Google Forms using a semi-structured, pretested questionnaire to identify their perception of research and publication. The questionnaire was formulated after a review of similar study materials and discussion with faculty and pilot-tested on a sample of 25 participants who were not included in the final study. Necessary amendments to the questionnaire were made based on the pilot study result. A Cronbach’s alpha was calculated for the questionnaire and found a value of 0.7 depicting satisfactory reliability. The questionnaire was forwarded to participants as a Google Form through WhatsApp and e-mail, and the survey was open from February 2024 to April 2024. The follow-up email/message was sent at monthly intervals of the survey window to encourage participation. The questionnaire contained data on participants’ characteristics (e.g., age and sex) and questions asked specifically about the student’s experiences and exposure to research and publication and perceived motivators and challenges associated with research and publication.

Ethical approval

Ethical approval was obtained from the Institutional Human Ethics Committee (IHEC), Baba Raghav Das (BRD) Medical College, Gorakhpur, before the commencement of the survey (35/IHEC/2024). Participation in this study was completely voluntary, and confidentiality and anonymity were assured. Informed consent was obtained from those who agreed to participate. A consent form was added at the beginning of the questionnaire explaining the purpose of the study and requesting their participation.

Statistical analysis

The data was downloaded from Google Forms in Microsoft Excel (Microsoft Corporation, United States), and a descriptive analysis was performed using IBM SPSS Statistics for Windows, version 22.0 (released 2013, IBM Corp., Armonk, NY). Quantitative variables were presented as means and standard deviation. Categorical variables were presented as numbers and percentages.

## Results

One hundred and ninety-six residents between the ages of 25 to 49 years participated in the study, where females outnumbered males. The mean age of the study participants was 30.64 ± 4.6 years. The sociodemographic profile of the study participants is given in Table 1. The majority of the participants were aged 30 years and below (63.3%), urban residents (76%), and married (60.2%).

**Table 1:** Sociodemographic characteristics of the participants (n = 196)

Sociodemographic profile		Number	Percentage
Age (years)	30 and below	124	63.3
	31–40	60	30.6
	40 and above	12	6.1
Gender	Male	93	47.4
	Female	103	52.6
Residence	Rural	47	24
	Urban	149	76
Marital status	Married	118	60.2
	Unmarried	78	39.8
Residency stage	First year	46	23.5
	Second year	70	35.7
	Third year	80	40.8

Table 2 shows the attitude of the participants toward research. The majority of the participants seemed interested in biomedical research (83.2) and thought that Basic Course in Biomedical Research (BCBR) helps conduct research projects (75%).

**Table 2:** Research attitude of the participants (n = 196)

Research attitude	Number of "Yes" responses (%)	Number of "No" responses (%)	Number of "Can’t say" responses (%)
Are you interested in biomedical research?	163 (83.2)	14 (7.1)	19 (9.7)
Are you willing to participate in a workshop on research methodology?	163 (83.2)	33 (16.8)	0
Are you willing to conduct clinical‑related research?	147 (75)	49 (25)	0
Are you willing to conduct community‑related research?	166 (84.7)	30 (15.3)	0
Do you think reading journals regularly will help in conducting biomedical research?	77 (39.3)	110 (56.1)	9 (4.6)
Do you think that Basic Course in Biomedical Research (BCBR) is helpful to PGs in conducting research?	147 (75)	42 (21.4)	7 (3.6)

The biomedical research experience of the participants is compiled in Table 3. Less than one-third of the residents had a publication in a journal.

**Table 3:** Research experience of the participants (n = 196)

Research experience	Number of "Yes" responses (%)	Number of "No" responses (%)
Have you participated in any research project previously?	108 (55.1)	88 (44.9)
Do you have any publications in a journal?	62 (31.6)	134 (68.4)
Have you presented a research paper at a conference?	97 (49.5)	99 (50.5)
Have you presented a poster at a conference?	142 (72.4)	54 (27.6)
Did you take the help of artificial intelligence in your research projects?	79 (40.3)	117 (59.7)

**Figure 1:**
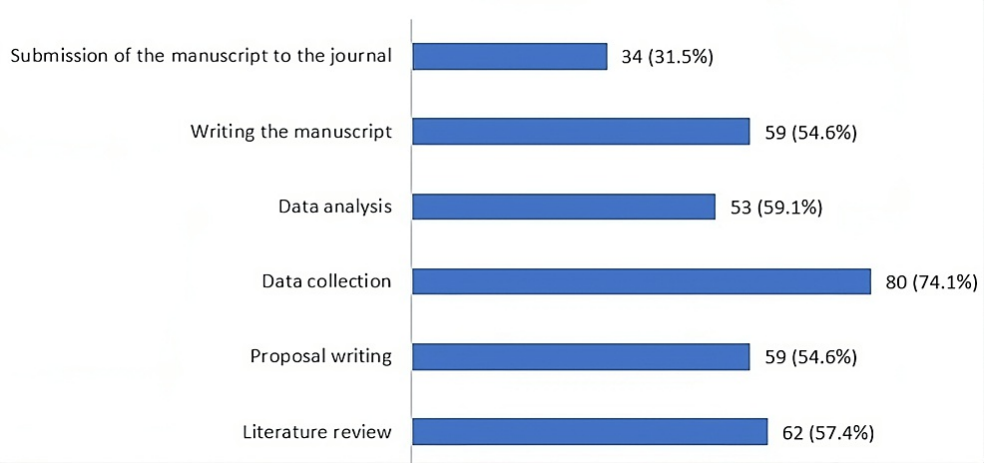
Past contribution of participants in the research project (n = 108; multiple responses possible)

**Figure 2:**
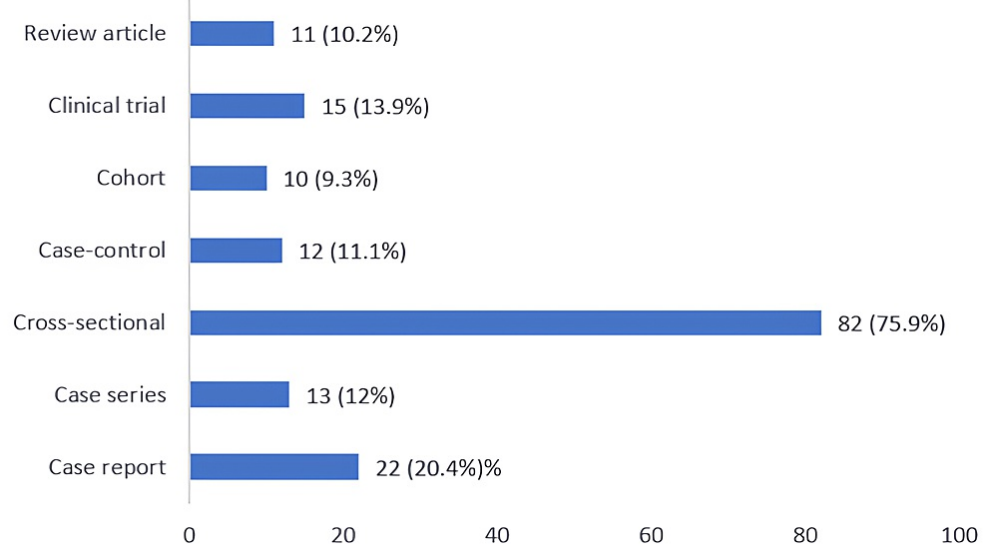
Type of research project conducted by the participants (n = 108; multiple responses possible)

The majority of the participants had contributed to data collection (74.1%) or had helped in the literature review (57.4%) in biomedical research (Figure 1). Cross-sectional study was the most commonly conducted (75.9%) study design by the participants (Figure 2).

The salient motivating factors for conducting the study among the participants are depicted in Table 4. Improving one’s research skills and desire to contribute to medical knowledge were found to be the most cited motivators for participating in research.

**Table 4:** Motivators for participating in research

Factors motivating to conduct research (multiple responses possible)	Number	%
Improve research skills	81	46.6
Desire to contribute to medical knowledge	80	46.0
Because it is compulsory (part of the curriculum)	71	40.8
Positive accomplishment on your resume	69	39.7
Fulfill your interest in research	62	35.6
To have your research published	56	32.2
Facilitate your acceptance into the residency program of choice	19	10.3

The lack of time due to being over‑burdened with academic and educational activity was found as the most common barrier to conducting research, followed by inadequate facility for research, as shown in Table 5.

**Table 5:** Barriers deterring from conducting research

Perceived barriers in conducting research (multiple responses possible)	Number	%
Lack of time due to being over‑burdened with academic and educational activity	94	48.5
Inadequate facility for research	62	32
Lack of statistical support	50	25.8
Difficulty in transportation	47	24.2
Inadequate support by mentors/assistants	46	23.7
Inadequate financial support	44	22.7
Difficulty in following up with the study subject (or patients)	43	22.2
Inadequate training in research during residency	42	21.6
Other personal commitments such as marriage, family	42	21.6
Lack of interest to the program/guide	42	21.6
Difficulty in publishing research during residency	42	21.6
Lack of reward and/or motivation	36	18.6
Lack of allocated research time	33	17
Lack of research curriculum	32	16.5
Difficulty in obtaining a research supervisor	29	14.9
Lack of interest	28	14.4
Poor accessibility to database (files)	26	13.4
Acknowledgment for contribution to research	21	10.8
Difficulty in presenting research during residency	20	10.3
Unavailability of the samples (or patients)	15	7.7

## Discussion

This study plays an important part in the exploration of the interest and determinants of research in community medicine postgraduate medical students. Research gives valuable insights into illness trends, risk factors, and intervention outcomes, enabling healthcare innovation [9,10]. Research provides the foundation for evidence-based medicine [11]. Researchers are thereby key in bridging the gap between current evidence and actual clinical practice. Research education and training facilities are essential for building a country's research infrastructure. The relative shortage of medical workforce trained in research methodology may hinder adequate research opportunities [12]. The Theory of Planned Behavior (TPB) is very interestingly coherent with our research question. The TPB highlights that an individual’s intentions to perform a behavior are the most immediate determinant of whether they will engage in that behavior [13].

The involvement of community medicine residents in research holds significant implications for both their academic and professional development. Engaging in research not only cultivates a spirit of inquiry and critical thinking but also exposes students to the latest advancements and challenges within their respective fields. Some institutions introduce formal research training during the medical undergraduate stage [14,15]. Researchers may also take the help of professional medical writers to make their study findings presentable [16]. The contribution of India is changing positively albeit at a slow pace. The contribution of India to the global research output in science, including health sciences, engineering, and other fields, was 3.12% in 2010, which increased to about 5.07% in 2020 [17].

In the present study, most of the community medicine residents were found interested in research projects. In our study, less than one-third of residents had a publication in a journal, which is similar to the findings of another study in India [18]. Although most of the participants were interested in the research activities, disproportionately less of them were involved in it. This is similar to the findings of previous studies [8,19]. In our study, the most common type of research was a cross-sectional study, which is in line with the study conducted by Soubhanneyaz et al., whereas another study found case reports as the main type of research in which residents had participated [20,21].

BCBR became compulsory for medical postgraduates admitted from July 2019 onwards as per the Medical Council of India (MCI) notice dated July 9, 2019 [17,22]. This foundational course equips students with the essential skills and knowledge necessary for conducting research in the medical field. Through a comprehensive curriculum covering research methodologies, literature review techniques, and ethical considerations, BCBR cultivates a research-oriented mindset among students, instilling in them the importance of evidence-based practice and scientific inquiry. By familiarizing students with the fundamentals of research design and data analysis, the course empowers them to critically evaluate existing literature, formulate research questions, and embark on independent research endeavors. Furthermore, the compulsory nature of BCBR ensures widespread exposure to research principles, fostering a culture of inquiry and scholarly activity within the postgraduate medical community. In our study, the majority of the participants thought that BCBR is helpful in research projects.

AI-driven tools are often used for data analysis, literature review automation, and even in clinical decision support systems. AI makes processing vast amounts of medical data easier, aiding students in their research endeavors by providing insights, identifying patterns, and assisting in hypothesis generation. In addition, AI-powered platforms offer personalized learning experiences, adapting to individual student needs and preferences, thereby enhancing research interest and productivity among postgraduate medical students. Moreover, AI catalyzes fostering interdisciplinary collaborations, enabling students to explore novel research domains and contribute to advancements in healthcare practices [23]. Less than half of the participants use AI for their research projects. Few previous studies also reflected the use of AI in research projects [24,25]. The scope and limitations need to be considered while using AI in medical research [26].

Desire to contribute to medical knowledge (46%), improve research skills (46.6%), mandatory in the curriculum (40.8%), and positive accomplishment on resume (39.7%) were some noticeable motivating factors. Leslie et al. also found that integrating research into residency training leads to a better understanding of research methodology, evidence-based medicine, successful publication, and improved job prospects [27].

Lack of time due to being preoccupied with other clinical and academic responsibilities was found as the leading hurdle in research engagement among the study population. This is in line with the study conducted by Gupta et al., where lack of time was found as a major obstacle for research by 88.3% of the residents [18]. Another study done by Sumi et al. also reported that too much paperwork was the most frequently cited obstacle in conducting clinical research, followed by lack of time [28]. The allied health professionals were found motivated to do research by intrinsic factors like a strong interest in research, while barriers include workload and lack of time [29].

By emphasizing the importance of research education and capacity building, the study reinforces the necessity of strengthening research infrastructure and promoting evidence-based medicine. Moreover, by bridging the gap between current evidence and clinical practice, healthcare workers can contribute to improving patient outcomes and healthcare delivery. Integrating research skills into medical education will cultivate a research-oriented mindset fostering critical thinking and problem-solving abilities, which are essential for addressing real-life clinical challenges to enhance evidence-based decision-making.

The curriculum should promote attending research methodology workshops focusing on developing research skills, critical appraisal abilities, and ethical research conduct. Establishing mentorship programs by joining residents with experienced researchers who can provide guidance, support, and encouragement throughout the research process may facilitate research culture among postgraduates. Recognizing the time constraints faced by residents and implementing strategies to optimize time management is an essential aspect to address. Decision-makers should invest in research infrastructure and facilities within community medicine departments to facilitate data collection, analysis, and dissemination. The institute should provide access to essential resources such as statistical support, research databases, and funding opportunities. Creating a cadre of healthcare workers with a research attitude and the application of knowledge gained should help low- and middle-income countries strengthen healthcare on their journey towards universal health coverage.

The study has a few limitations. Reliance on an online survey may introduce sampling bias, as it only captures responses from participants who have Internet access and are willing to participate. Self-reported data, misinterpretation of questions, and recall bias may be additional sources of bias. The cross-sectional study design fails to give causality.

## Conclusions

The majority of community medicine residents were found interested in research activities and participated in some sort of research activity in the past. The majority of the participants thought that BCBR is helpful in research projects and a considerable proportion of them use AI for their research projects. The opportunity to improve research skills, desire to serve the medical fraternity through research, and a positive impact on resumes were the leading motivating factors for conducting research. Difficulty in sparing time, little knowledge, and poor support from mentors were found as important barriers. Addressing these barriers through effective curriculum design may encourage more residents to participate in research activities thereby improving their research skills and serving mankind.
